# Behavioural and emotional comorbidities in school-aged children with neurological conditions in Kilifi, Kenya, and their long-term consequences

**DOI:** 10.1080/16549716.2022.2034132

**Published:** 2022-02-09

**Authors:** Judy K. Baariu, Symon M Kariuki, Charles RJC Newton

**Affiliations:** a Clinical Research-Neurosciences Department, KEMRI/Wellcome Trust Research Programme, Centre for Geographic Medicine Research-Coast (Cgmrc), Kilifi, Kenya; b Department of Public Health, School of Human and Health Sciences, Pwani University, Kilifi, Kenya; c Department of Psychiatry, University of Oxford, Oxford, UK

**Keywords:** Behavioural problems, emotional problems, mental health problems, children, neurological conditions

## Abstract

**Background:**

Neurological conditions and mental health problems are common in children in low- and middle-income countries, but the risk factors and downstream impact of these problems on children with neurological conditions are not reported.

**Objective:**

To determine the association of neurological conditions with behavioural and emotional problems in children, the prevalence and risk factors of behavioural and emotional problems, and long-term impact of these conditions.

**Methods:**

Data on multiple neurological conditions and mental health problems were available for 1,616 children (aged 6–9 years) from Kilifi, Kenya. Neurological conditions were diagnosed using standardised tools and clinical examination. Behavioural and emotional problems assessed using Child Behaviour Questionnaire for Parents. Long-term outcomes were obtained from census data of the Kilifi Health and Demographic Surveillance System. Logistic and linear regression were used to measure associations.

**Results:**

Mental health problems were higher in those with any neurological condition compared to those without (24% vs. 12%, p < 0.001). Cognitive (odds ratio (OR) = 2.39; 95% CI: 1.59–3.59), motor (OR = 3.17; 95% CI: 1.72–5.82), hearing (OR = 2.07; 95% CI:1.12–3.83) impairments, and epilepsy (OR = 4.18; 95% CI: 2.69–6.48), were associated with mental health problems. Prevalence of any mental health problem was 15%, with externalizing problems more common than internalizing problems (21% vs. 17%, p = 0.004). Longitudinal follow-up indicated that the disorders affected an individual’s future schooling (e.g. OR = 1.25; 95% CI: 0.14–1.46 following cognitive impairments), occupation (OR = 2.44; 95% CI: 1.09–5.44 following mental health problems), and access to household assets (OR = 2.78; 95% CI: 0.99–7.85 following epilepsy).

**Conclusions:**

Neurological conditions in school-aged children in Kilifi are associated with mental health problems, and both disorders have long-term consequences. Preventive and therapeutic measures for these conditions are needed to improve outcomes of these children.

## Background

Neurological conditions contribute a substantial proportion of the global burden of diseases, in terms of disability-adjusted life years [1]. The global burden of these conditions may have decreased over the last two decades but remain an important public health problem in low- and middle-income countries (LMICs). In LMICs, many children are exposed to multiple risk factors for neurological conditions, including poverty, poor health, lack of adequate nutrition, and unstimulating home environments [2], which detrimentally affect their functional capacity [3]. Neurological conditions in children have been associated with medical and psychiatric comorbidities [4–6]. These comorbidities have a significant impact on children and younger adolescents [7] due to the large burden of disability and mortality associated with them [8].

Neurological conditions are due to damage or anomaly of the nervous system, resulting in abnormal body structure or functioning. These conditions can manifest as impairments of different components of functioning such as motor function, hearing, vision, cognition (referred to here as the inability to process, learn, and remember information) [9], and enduring susceptibility to unprovoked seizures i.e. epilepsy. Emotional and behavioural disorders (classified as either ‘internalizing’ or ‘externalizing’ respectively, and/or ‘mental health problems’ collectively [10]), co-exist with childhood neurological conditions and both can have a significant negative impact on the wellbeing of the child and family due to challenges such as stigma, discrimination, and social isolation. In LMICs, these psychosocial challenges are further compounded by poverty, lack of support and services, and hostile environments. In the long term, children with disabilities will be poorer on a host of measures including less access to education, poor health-care services, and low socioeconomic status [11].

While multiple neurological conditions in childhood have been studied in LMICs [12,13], there are no data on their comorbidity with behavioural and emotional problems in school-aged children and the long-term consequences of these disorders. A study carried out in rural Kilifi, Kenya reported a significant burden of multiple neurological conditions in the population, with 6% of the older children having moderate or severe impairments [9]. This study found that cognitive, hearing impairments, and epilepsy were most common, with 22% found to have more than one neurological condition [9]. This study, however, did not report the prevalence of mental health conditions, their association with these neurological conditions, and their long-term impact on functioning capacity in society.

Evidence on the associations between neurological conditions and mental health problems, and their impact on future functioning is of public health importance, as it will influence the development of comprehensive packages of care that advocate for early detection, management, and support of children with these conditions and problems. We determined the prevalence and risk factors for behavioural and emotional problems in school-aged children with neurological conditions and investigated their associations with visual, motor, cognitive, hearing impairments, and epilepsy. Also, leveraging on a demographic surveillance system, we sought to evaluate the long-term educational and socio-economic developmental impact of the mental health problems and neurological conditions.

## Methods

### Study setting and participants

An epidemiological survey was carried out between June 2001 to March 2002 on neurological disabilities, impairments, and mental health problems in children aged 6–9 years in a rural area in Kilifi County [9]. The county is located along the coast of Kenya and the area residents are mainly Mijikenda, a Bantu group of nine tribes with the Giriama dominating. Kilifi County has a population distribution of 1.5 million residents, whereby 0–14 year olds constitute 42% of the total population [14].

In this study, we selected children aged 6 years and above owing to the difficulty in identifying and assessing mental and neurological disorders in younger children especially hearing, visual, and cognitive impairments. Additionally, having survived early childhood, which is a period associated with high adversity and mortality in sub-Saharan Africa [15], studying children above 6 years of age enabled understanding the impact of early-life negative impacts of neurological conditions, including being able to access education.

### Assessment of neurological conditions and mental health problems

Assessments were performed in two stages during the 2001 epidemiological survey (Figure S1). To screen for neurological impairment and epilepsy in stage I, five trained field interviewers fluent in the local Kigiryama language administered Ten Questions Questionnaire (TQQ) [16], to parents or guardians of 10,218 children who agreed to participate. The TQQ consists of ten items (with a yes or no response) designed to detect moderate-to-severe impairments and disorders; including five questions addressing cognitive development, two questions relating to motor ability, and one question each regarding vision, hearing, and seizures. Those who tested positive on the TQQ, and a random sample of those who tested negative (10.3%), were invited to participate in stage II. In this stage, a team of clinicians and psychological assessors performed clinical history, examination, and psychological assessments to detect cognitive, motor, hearing, visual impairments, and epilepsy (Table S1). Perinatal and postnatal adverse occurrences were documented from medical history and maternal recall of pregnancy and delivery events, a method that was shown to be relatively reproducible and accurate in previous reports [17,18]. Data on perinatal events included pregnancy complications, place and mode of delivery, and birth trauma/difficulties, while postnatal occurrences assessed were history of neonatal insults, neonatal jaundice, developmental problems, child’s immunization, and neurological deficit.

During assessments in stage II, a Child Behaviour Questionnaire for Parents (CBQFP) was administered to assess behavioural and emotional problems in the children. The CBQFP was administered to a parent or guardian in a conversational manner comprising of 15 items to assess various aspects of behaviour and emotion including reaction to change, independence, mood, worries, fears, and habits. The severity and frequency of behaviour described in the questionnaire were rated and scored depending on the parents’ response with a higher overall score signifying a higher level of total behaviour or emotional problems [19]. The CBQFP had been previously adapted and validated for use in this setting, demonstrating a high degree of interrater reliability (*r* = 0.92), and fair internal reliability (standardized item α = 0.61) in the assessment of neuropsychological outcomes of cerebral malaria [19].

Questions in the CBQFP on anxiety, temper, mood, worries, fears, and empathy were classified as indicators for internalizing or emotional problems; while those on the child’s concentration span, social relationships, social dependency, and behaviour in public assessed for externalizing problems. Assessment data from questions on appetite, habits, self-care, and wetting/soiling oneself were not included in the analysis as they were not categorized as indicators for internalizing or externalizing problems.

### Long-term follow-up of study participants

We followed up the children assessed in stage II through the Kilifi Health and Demographic Surveillance System (KHDSS) from June 2001 to May 2008, to assess any long-term consequences of neurological conditions, behavioural and emotional problems on their education and socio-economic status. The KHDSS is a database that was established to create longitudinal community-based records of births, deaths, pregnancies, migration events, and additional sociodemographic information including socio-economic status and educational achievement [20]. The surveillance region includes an estimated population of 280,000 residents [21] living in an area covering 891 km^2,^ which is in reference to the area served by the county’s main referral hospital.

Follow-up of the participants was done through 4-monthly household visits during which data on their schooling, education level and years completed, economic status (measured by access to a source of lighting), assets acquired (e.g. a working mobile phone, radio) at a household level, and occupation were collected by trained field workers using standardised data collection tools.

### Statistical analysis

All statistical analyses were performed using STATA version 15 (StataCorp, College Station, TX, USA). Demographic characteristics between those who tested positive or negative on the TQQ test were compared using Pearson χ^2^ test (for categorical measures) and Student’s t-test (for continuous measures). Associations between neurological conditions and total mental health problems were illustrated using Pearson χ^2^ test, while Fisher’s exact test was used for comparison of infrequent observations [22]. The associations were further evaluated through building of age and sex adjusted linear and logistic regression models.

The cut-off score for behavioural and emotional problems was derived from the 90^th^ percentile of total behavioural and emotional scores among children who screened negative for neurological conditions, with the resultant cut-off of the mental health problems applied to all participants in the dataset. This was done following guidelines provided by Richman *et al* [23], and similar criteria were used to derive cut-offs for internalizing and externalizing problems, respectively.

Age and sex stratified prevalence of total behavioural and emotional problems were computed, and linear regression applied to identify significant risk factors for total mental health problems scores, total internalizing, and externalizing scores. A logistic regression model was also used to determine potential risk factors associated with internalizing and externalizing problems as categorical variables, and in determining long-term impact of neurological conditions and mental health problems on schooling, occupation, and asset ownership. Risk factors with a univariable p ≤ 0.25 were fitted into a sex and age adjusted multivariable linear and logistic regression model to further identify independent factors.

## Results

In stage I, of the 10,218 children assessed using the TQQ, 955 (9.3%) screened positive. In stage II, the CBQFP was administered to caregivers of 716 children who screened positive on the TQQ and 900 of those who screened negative (Figure S1). These children were also clinically assessed for various neurological conditions. A total of 1,616 children were assessed in stage II with 51.1% being male. The participants had a mean age of 7.4 (1.12 SD) years.

### Association between neurological conditions and mental health problems

Adjusted for attrition and sensitivity of the screening tool, the prevalence of a neurological condition in the sample population was 61/1000, the most common domains being cognitive impairment (31/1000), epilepsy (41/1000), and hearing impairment (14/1000). Motor and visual impairments occurred in 5/1000 and 2/1000 of the children, respectively [9].

Total behavioural and emotional problems occurred in 23.9% of children with neurological conditions, compared with only 12.1% of children without (p < 0.001). In a univariable model adjusted for age and sex, cognitive (odds ratio (OR) = 2.39; 95% confidence intervals (95% CI): 1.59–3.59), motor (OR = 3.17; 95% CI: 1.72–5.82), hearing (OR = 2.07; 95% CI:1.12–3.83) impairments, and epilepsy (OR = 4.18; 95% CI: 2.69–6.48), were associated significantly with behavioural and emotional problems (Table 1).

**Table 1.** Association of neurological conditions with total behavioural and emotional problems

Neurological conditions	Adjusted OR (95% CI)	P-value	Adjusted β coefficient (95% CI)	P-value
Cognitive impairment	2.39 (1.59–3.59)	<0.001	1.28 (0.19, 2.37)	0.021
Visual impairment	1.13 (0.24–5.27)	0.877	−0.05 (−3.51, 3.42)	0.980
Hearing impairment	2.07 (1.12–3.83)	0.020	0.39 (−1.22, 2.02)	0.631
Motor impairment	3.17 (1.72–5.82)	<0.001	2.11 (0.31, 3.91)	0.022
Epilepsy	4.18 (2.69–6.48)	<0.001	1.62 (0.33, 2.92)	0.014
Any neurological condition	2.20 (1.59–3.04)	<0.001	0.85 (0.06, 1.64)	0.030

Internalizing problems were also more common in children with neurological conditions (23.2%) than those without (14.8%). Following an adjusted univariable logistic model, cognitive (OR = 1.58; 95% CI: 1.05–2.38), hearing (OR = 2.14; 95% CI:1.23–3.73), motor (OR = 2.87; 95% CI:1.59–5.15) impairments, and epilepsy (OR = 2.75; 96% CI:1.78–4.24) were associated with total internalizing problems (Table 2). While motor impairment (OR = 2.23; 95% CI: 1.23–4.03), epilepsy (OR = 3.43; 95% CI: 2.26–5.02), and cognitive impairment (OR = 2.16; 95% CI: 1.48–3.14) were particularly associated with externalizing problems (Table 2).

**Table 2.** Association of neurological conditions with internalizing and externalizing problems

Neurological conditions	Internalizing problems		Externalizing problems	
	Adjusted OR (95% CI)	Adjusted β coefficient (95% CI)	Adjusted OR (95% CI)	Adjusted β coefficient (95% CI)
Cognitive impairment	1.58* (1.05–2.38)	0.45** (0.26, 0.65)	2.16** (1.48–3.14)	0.25 (−0.78, 1.28)
Visual impairment	0.89 (0.19–4.00)	−0.26 (−0.85, 0.33)	1.32 (0.36–4.85)	−0.19 (−3.52, 3.13)
Hearing impairment	2.14** (1.23–3.73)	0.28* (−0.01, 0.56)	1.35 (0.74–2.46)	−0.13 (−1.67, 1.41)
Motor impairment	2.87** (1.59–5.15)	0.68** (0.35, 1.01)	2.23** (1.23–4.03)	0.66 (−1.04, 2.37)
Epilepsy	2.75** (1.78–4.24)	0.72** (0.48, 0.95)	3.43** (2.26–5.20)	0.42 (−0.81, 1.65)
Any neurological condition	1.68** (1.23–2.30)	0.39** (0.24, 0.55)	1.79** (1.33–2.42)	0.07 (−0.67, 0.81)

*p-value<0.05; **p-value<0.01

### Prevalence of behavioural and emotional problems

The prevalence of total behavioural and emotional problems in the sample population was 15.3% (95% CI: 13.5–17.1). Additionally, the prevalence of internalizing and externalizing problems was 16.9% (95% CI: 15.1–18.7) and 20.8% (95% CI: 18.9–22.8), respectively, with the latter more prevalent than the former (p = 0.004).

Following stratification by age, the prevalence of behavioural and emotional problems was highest among the younger children (21.7% (95% CI: 18.0–25.4)) and declined with age with the lowest prevalence at 9 years (10.1% (95% CI: 6.9–13.3)). Male children had more mental health problems compared to females (p < 0.001) (Table S2).

### Risk factors for behavioural and emotional problems

In an univariable linear regression, history of neonatal jaundice (β = 2.01; 95% CI: 0.91, 3.10), neurological deficit (β = 2.12; 95% CI: 1.38, 2.87), cognitive (β = 1.29; 95% CI: 0.27, 2.33), and motor impairments (β = 2.09; 95% CI: 0.42, 3.78) were significantly associated with total behavioural and emotional scores (Table 3). These factors were consistently identified as important risk factors for total behavioural and emotional problems in a univariable logistic model. In a multivariable logistic regression adjusted for age and sex; neonatal jaundice (OR = 2.28; 95% CI: 1.38–3.75) remained significantly associated with the mental health problems (Table S3).

**Table 3.** Risk factors for total behavioural and emotional problems and scores

Risk factors	Binary total behavioural and emotional problems		Continuous total behavioural and emotional scores	
	OR (95% CI)	P-value	β coefficient (95% CI)	P-value
**Sociodemographic factors**				
Younger age (6–7 years)	1.79 (1.34–2.39)	<0.001	0.49 (−0.09, 1.08)	0.101
Older age (8–9 years)	0.56 (0.42–0.74)	<0.001	−0.49 (−1.08, 0.09)	0.101
Male	1.69 (1.28–2.24)	<0.001	0.14 (−0.45, 0.73)	0.641
Number of children in the family	0.99 (0.94–1.05)	0.912	0.13 (0.01, 0.25)	0.043
Poor nutritional status	0.76 (0.41–1.42)	0.393	−0.01 (−1.23, 1.22)	0.996
Child not schooling	1.37 (1.05–1.80)	0.02	0.93 (0.34, 1.52)	0.002
Father’s lack of economic activity	1.16 (0.84–1.59)	0.375	−0.01 (−0.79, 0.79)	0.991
Mother’s lack of economic activity	1.15 (0.85–1.55)	0.360	0.18 (−0.48, 0.84)	0.599
Single maternal marital status	0.79 (0.55–1.14)	0.210	−0.45 (−1.22, 0.31)	0.246
No maternal education	0.67 (0.50–0.89)	0.005	0.04 (−0.59, 0.68)	0.898
**Perinatal factors**				
Home delivery	0.75 (0.53–1.07)	0.112	−0.28 (−1.07, 0.52)	0.497
Birth difficulty	1.54 (0.97–2.45)	0.068	0.52 (−0.60, 1.65)	0.361
**Post-natal factors**				
Neonatal insults	0.87 (0.29–2.54)	0.799	0.36 (−1.12, 1.85)	0.631
Child not immunized	0.86 (0.45–1.64)	0.645	−0.44 (−2.25, 1.36)	0.630
Neonatal jaundice	2.52 (1.66–3.81)	<0.001	2.01 (0.91, 3.10)	<0.001
Neurological deficit	3.38 (2.13–5.36)	<0.001	2.12 (1.38, 2.87)	<0.001
Developmental problems	1.98 (1.40–2.78)	<0.001	0.76 (−0.09, 1.61)	0.079
**Neurological conditions**				
Cognitive impairments	2.42 (1.64–3.57)	<0.001	1.29 (0.27, 2.33)	0.013
Hearing impairments	1.66 (0.92–3.00)	0.092	0.23 (−1.27, 1.73)	0.768
Visual impairments	0.89 (0.20–4.01)	0.889	−0.24 (−3.39, 2.91)	0.881
Motor impairments	3.11 (1.73–5.58)	<0.001	2.09 (0.42, 3.78)	0.014
Epilepsy	3.88 (2.55–5.90)	<0.001	1.58 (0.38, 2.78)	0.010

Internalizing scores were associated with birth difficulties (β = 0.43; 95% CI: 0.19, 0.66), history of neonatal jaundice (β = 0.41; 95% CI: 0.18, 0.63), and repetitive unprovoked seizures (β = 0.69; 95% CI: 0.46, 0.93). These factors were also identified as risk factors for binary internalizing problems following a logistic regression model, as well as motor impairments (OR = 2.86; 95% CI: 1.16–5.09), developmental problems (OR = 1.53; 95% CI: 1.09–2.14), and neurological deficits (OR = 2.76; 95% CI: 1.74–4.38) (Table 4). In an adjusted multivariable model, neurological deficits, history of neonatal jaundice, and epilepsy maintained as significant covariates for total internalizing problems (Table S3).

**Table 4.** Risk factors for total internalizing and externalizing problems and scores

Risk factors	Internalizing problems and scores		Externalizing problems and scores	
	Univariable OR (95% CI)	Univariable β coefficient (95% CI)	Univariable OR (95% CI)	Univariable β coefficient (95% CI)
**Sociodemographic factors**				
Younger age (6–7 years)	1.51** (1.15–1.97)	0.17** (0.05, 0.29)	1.43** (1.12–1.84)	−0.09 (−0.64, 0.46)
Older age (8–9 years)	0.66**(0.51–0.87)	−0.17** (−0.29, -0.05)	0.69** 0.54–0.89	0.09 (−0.46, 0.64)
Male	1.14 (0.88–1.49)	0.04 (−0.08, 0.16)	1.53** (1.19–1.95)	−0.06 (−0.60, 0.48)
Number of children born to the family	1.03 (0.98–1.09)	0.02 (−0.01, 0.05)	1.01 (0.96–1.06)	0.09 (−0.02, 0.21)
Poor nutritional status	0.88 (0.49–1.55)	0.09 (−0.16, 0.34)	0.84 (0.49–1.43)	−0.22 (−1.35, 0.91)
Father’s lack of economic activity	1.06 (0.78–1.45)	0.12 (−0.04, 0.27)	1.11 (0.83–1.48)	−0.26 (−1.01, 0.48)
Mother’s lack of economic activity	1.19 (0.89–1.59)	0.08 (−0.05, 0.21)	1.18 (0.90–1.54)	0.08 (−0.54, 0.69)
Single maternal marital status	0.98 (0.70–1.36)	−0.07 (−0.22, 0.09)	0.75 (0.54–1.03)	−0.33 (−1.04, 0.38)
No maternal education	0.73* (0.56–0.95)	−0.07 (−0.20, 0.05)	0.64** (0.49–0.82)	−0.07 (−0.67, 0.51)
**Perinatal factors**				
Home delivery	0.85 (0.60–1.19)	−0.07 (−0.23, 0.10)	0.79 (0.57–1.08)	−0.29 (−1.03, 0.45)
Birth difficulty	1.74** (1.13–2.69)	0.43** (0.19, 0.66)	1.25 (0.81–1.94)	−0.13 (−1.17, 0.92)
**Post-natal factors**				
Neonatal insults	0.78 (0.27–2.28)	−0.23 (−0.89, 0.44)	1.21 (0.45–3.21)	0.37 (−0.18, 0.92)
Child not immunized	1.09 (0.62–1.92)	0.01 (−0.25, 0.27)	0.64 (0.35–1.15)	−0.64 (−2.39, 1.11)
Neonatal jaundice	1.65* (1.07–2.55)	0.41** (0.18, 0.63)	2.67** (1.82–3.92)	1.09* (0.07, 2.11)
Neurological deficit	2.76** (1.74–4.38)	0.79** (0.52, 1.05)	2.47** (1.57–3.87)	0.78** (0.22, 1.33)
Developmental problems	1.53** (1.09–2.14)	0.32** (0.15, 0.49)	1.87** (1.36–2.56)	0.10 (−0.69, 0.89)
**Neurological conditions**				
Cognitive impairment	1.58* (1.06–2.36)	0.45** (0.26, 0.65)	2.19** (1.52–3.15)	0.23 (−0.74, 1.19)
Hearing impairment	1.92* (1.11–3.32)	0.20 (−0.09, 0.49)	1.17 (0.65–2.09)	−0.15 (−1.55, 1.24)
Visual impairment	0.77 (0.17–3.42)	−0.35 (−0.97, 0.28)	1.06 (0.29–3.79)	−0.22 (−3.17, 2.2)
Motor impairment	2.86** (1.61–5.09)	0.68** (0.35, 1.01)	2.21** (1.24–3.95)	0.65 (−0.92, 2.22)
Epilepsy	2.68** (1.75–4.09)	0.69** (0.46, 0.93)	3.32** (2.21–4.97)	0.42 (−0.71, 1.54)

*p-value<0.05; **p-value<0.01

Neurological deficit was related as a risk factor for total externalizing scores (β = 0.78; 95% CI: 0.22, 1.33). Male sex (OR = 1.53; 95% CI: 1.19–1.95), lack of maternal education (OR = 0.64; 95% CI 0.49–0.82), and history of neonatal jaundice (OR = 2.67; 95% CI: 1.82–3.92) were identified as significant risk factors for externalizing problems (Table 4). Using an adjusted multivariable model, neonatal jaundice (OR = 3.66; 95% CI: 1.78–7.55), neurological deficit (OR = 3.30; 95% CI: 1.27–8.58), and repetitive unprovoked seizures (OR = 2.87; 95% CI: 1.45–5.68) remained as significant determinants of externalizing problems in the study population. The presence of neurological deficit was also highly correlated with the presence of total externalizing score (β = 0.54; 95% CI: 0.10, 0.97); pseudo-R2 = 0.12) in a multivariable linear regression model (Table S3).

### Long-term impact of neurological conditions and mental health problems

Of the 1,616 children assessed, 908 (56.2%) completed the follow-up including those who screened positive on the TQQ (393 [54.9%] of 716) and (515 [57.2%] of 900) who screened negative. Adjusted univariable models demonstrated that cognitive (OR = 1.25; 95% CI: 0.14–1.46) and hearing (OR = 1.42; 95% CI: 0.18–1.99) impairments, had significant impact on an individual’s schooling. Epilepsy status assessed previously was also significantly associated with risk for poor outcome in terms of the assets acquired over time (OR = 2.78; 95% CI: 0.99–7.85) (Table 5).

**Table 5.** Age and sex adjusted analysis of long-term impact of neurological conditions and mental health problems

Predictors of outcome	Occupation Adjusted OR (95% CI)	Schooling Adjusted OR (95% CI)	Access to modern source of lighting Adjusted OR (95% CI)	Assets; working mobile phone or radio Adjusted OR (95% CI)
**Neurological conditions**				
Cognitive impairment	1.20 (0.40–3.58)	1.25** (0.14–1.46)	0.39* (0.17–0.92)	1.63 (0.79–3.36)
Epilepsy	1.78 (1.59–5.36)	1.40 (0.53–3.69)	1.71 (0.89–3.31)	2.78* (0.99–7.85)
Hearing impairment	1.46 (0.33–6.53)	1.42* (0.18–1.99)	0.53 (0.16–1.77)	0.79 (0.34–1.86)
Visual impairment	-	0.22 (0.03–1.49)	-	1.03 (0.12–8.88)
Motor impairment	0.71 (0.09–5.59)	0.45 (0.16–1.23)	0.70 (0.21–2.38)	1.61 (0.48–5.44)
**Mental health problems**				
Total behaviour and emotional problems	2.44* (1.09–5.44)	1.56* (1.32–1.98)	1.26 (0.77–2.07)	1.29 (0.76–2.22)
Internalizing problems	0.99 (0.41–2.45)	0.83 (0.48–1.43)	1.27 (0.80–2.02)	1.71* (1.00–2.95)
Externalizing problems	1.85 (0.91–3.76)	1.57* (1.35–1.90)	1.73** (1.15–2.62)	1.11 (0.71–1.72)

*p-value<0.05; **p-value<0.01

In an adjusted logistic model, screening positive for behavioural and emotional problems affected an individual’s schooling (OR = 1.56; 95% CI: 1.32–1.98), and their employment (OR = 2.44; 95% CI: 1.09–5.44). Those with internalizing problems had less access to household assets (OR = 1.71; 95% CI: 1.00–2.95) such as a mobile phone or working radio over time while externalizing problems significantly affected an individual’s and family socioeconomic outcomes including access to a modern source of lighting (OR = 1.73; 95% CI: 1.15–2.62) such as electricity (Table 5).

## Discussion

This study has provided evidence that neurological conditions are significantly associated with behavioural and emotional problems in the cross-sectional component of the study, and both conditions occur concomitantly in over a fifth of children. Up to 15% of the children experienced a mental health problem, with externalizing problems greater than internalizing problems. The risk factors for these problems included neurological deficits, neonatal jaundice, and repetitive unprovoked seizures. The long-term longitudinal follow-up component of the study demonstrated that mental health problems and neurological conditions had a negative impact on future schooling, employment, and economic status of these children, necessitating institution of measures and policies to support them throughout life.

### Association between neurological conditions and mental health problems

The prevalence estimates of the different neurological conditions for this sample population have been previously reported by Mung’ala-Odera *et al*, who identified a significant burden (61/1000) of moderate or severe neurological conditions in the Kenyan children [9]. This prevalence is comparable with estimates reported in South Africa (60/1000) [24] but is higher compared to findings from Ghana (16.6/1000) [25], and from a community survey of neurological conditions in children conducted in India, which documented a prevalence of 31.3 per 1000 children [26].

We found an association of the various neurological conditions with behavioural and emotional problems. The mental health problems were identified in 24% of children with neurological conditions compared with only 12% healthy controls. Mental health outcomes following neurological exposures may have been higher as the TQQ only reliably detects moderate-to-severe impairment and not mild neurological impairments, which are associated with mental health problems. Alternatively, some associations could have been underestimated as the TQQ has a low sensitivity in detecting vision and hearing impairments [27].

Cumulatively, neurological conditions demonstrated significant association with mental health problems, and the availability of individual neurological problems allowed further assessment of their relative contribution to the association. Clinically defined cognitive, motor impairments, and epilepsy were significantly associated with internalizing and externalizing problems in the children. These findings are similar to those from previous studies conducted in LMICs, which demonstrated association between epilepsy and internalizing and externalizing problems, often related to early onset of epilepsy and frequent seizures [28,29]. As in our study, King-Dowling *et al* found that young children with motor difficulties such as developmental coordination disorder tend to have more emotional-behavioural problems when compared to their typically developing peers [6].

The finding that multiple neurological conditions were associated with behavioural and emotional problems demonstrates their interaction within a broad spectrum of neurodevelopmental disorders. Few studies in LMICs have examined associations of many neurological conditions together with behavioural and emotional problems and therefore these analyses add important information to the literature.

Associations between neurological conditions and mental health problems are of public health interest as it could likely influence development of comprehensive packages of care for detection and management of all these problems in children presenting to health facilities. There is a need to develop more sensitive and easier to administer tools for detecting impairments such as vision and hearing. The associations of mental health problems and neurological conditions are based on cross-sectional measurements, and it is difficult to infer causality direction, necessitating the need for future longitudinal follow-up studies.

### Prevalence of behavioural and emotional problems

The prevalence estimates for all mental health problems in this study (15%) are comparable to results in two Kenyan studies conducted on school-aged children and adolescents (17%) [30] and in younger children (13%) [31]. When stratified by age, the burden of mental health problems was significantly higher in younger children as compared to older children. This finding suggests a possible remission of behavioural and emotional problems with age, but also shows that even in children of older ages in this study, levels of mental health problems are high. Externalizing problems were more prevalent than internalizing problems across all the stratified age groups. These could partly be attributed to the sensitivity of the CBQFP in identifying and detecting externalizing problems more since the questions for the former problems were comparatively many. The relatively increased prevalence of externalizing problems also implies the increased manifestation of these problems, similar to attention deficit hyperactivity disorder [32]. Stratified prevalence of total behavioural, and externalizing problems were significantly higher among male children compared to the females similar to other studies [33,34].

### Risk factors for behavioural and emotional problems

History of neonatal jaundice, presence of neurological deficits, and epilepsy were consistently identified as some of the key risk factors for behavioural and emotional problems. Various studies have demonstrated an association between neonatal jaundice and neurological damage [35,36]. Several LMICS have also reported high rates of bilirubin-induced neurodevelopmental disorders and/or neurological impairments [37–39], which can subsequently manifest as behavioural or emotional problems [40]. A prospective cohort study carried out in Finland reported an association between hyperbilirubinemia and neurobehavioral problems such as attention deficit hyperactivity disorder (ADHD) in children [41]. A systematic review by Mwaniki *et al* however showed that many studies do not document behavioural and emotional consequences of neonatal jaundice in LMICs and new data are therefore needed [42] to add to the current evidence. Preventive and management interventions for neonatal jaundice in LMICs need to be scaled up through strengthening health-care systems and include treating neonatal sepsis early, detection of potential haemolytic causes, effective phototherapy, and having blood available for exchange transfusion [38,39]. Future prospective studies are also required to determine long-term outcome of these interventions.

Identification of epilepsy as a risk factor for behavioural or emotional problems in this study is analogous to studies from similar settings such as Congo and Tanzania, where reported behavioural problems occurred in 29% and 66% of children with epilepsy, respectively, [29,43]. Epilepsy is a chronic neurological disorder whose association with behavioural and emotional problems can be related to the underlying neurological damage, shared risk factors and genetic susceptibility, or damage from seizures themselves [5]. Recent studies have shown that even febrile seizures and acute symptomatic seizures that were previously thought to have favourable outcomes are also associated with increased prevalence of behavioural and emotional problems [44].

### Long-term impact of neurological conditions and behavioural/emotional problems

Hearing, cognitive impairments, and epilepsy had a downstream negative impact on the individual’s ability to acquire jobs, on their schooling, and acquisition of assets at a household level. As expected, scholastic achievement was particularly affected by cognitive impairment, which can cause inability to process, learn, and remember information, and by hearing in which inability to hear sounds or hear oneself affects learning of reading, writing, and social skills. Epilepsy was associated with fewer assets, likely related to family’s low socioeconomic status and unemployment due to stigma from seizures. The outcomes were particularly worse when neurological conditions and mental health problems co-occurred in one child, supporting the added burden of comorbid conditions. Behaviour and emotional problems likely affect the child’s concentration in school affecting their grades, leading to eventual dismissal or drop-out from school. Some neurological impairments such as cognition affect memory, learning, and reasoning, directly affecting the schooling process.

The long-term consequences could also be due to the stigma and discrimination associated with these conditions especially hearing and motor deficits which will see many children affected ostracized resulting in them dropping out of school. One problem then leads to another with poor education leading to the unemployment status, but stigma may also play a role in finding a job for the person with the disorder and family members or caregivers. Associations with poor quality of life are not surprising as this has consistently been demonstrated in many studies [45,46]. These findings support the need to find early interventions that will not only improve the quality of life of people living with these disorders and that of their families but also ease their integration into the community. In particular, there is a need to set up interventions such as occupational therapies (to overcome any barriers that prevent them from daily activities of living), speech and language therapy (to promote communication or eating, drinking and swallowing), and physiotherapies (to promote, develop or restore physical movement and strength) [47,48].

## Strengths and limitations

The strength of this study is that the estimates of the association between neurological conditions and behavioural and emotional problems are based on a community sample, which is not subject to severity bias compared to a hospital sample. The sample size was large for most of the conditions, and tools adapted to the local populations were used to conduct the assessments. The long-term outcomes of these disorders were done prospectively which can reliably establish direction of the association. The limitations included the cross-sectional component of the study which could not infer causality between neurological conditions and mental health problems. There were small numbers of children with visual and hearing impairments probably related to the insensitivity of the screening tool, i.e., TQQ. The TQQ only reliably detects moderate-to-severe impairment and not mild impairments, possibly underestimating the number of children with disability.

## Conclusions

Multiple neurological conditions are associated with behavioural and emotional problems in school-going children. Some risk factors for mental health problems in children such as neonatal jaundice and seizures can be prevented through strengthening health-care systems to prevent and manage these conditions. Additionally, mental and neurological conditions may have long-term negative impact on schooling, employment, and economic status, and therefore their early identification and management can lead to improved long-term outcomes. Future longitudinal studies are required to establish the causality direction between neurological conditions and behavioural and emotional problems. Quality care, cost-effective interventions, and vigilant follow-ups are also needed in LMICs to ensure that these children survive and thrive, and to reduce the burden of these disorders on these children and their families.

## Supplementary Material

Supplemental Material

## Paper context

Neurological conditions are common in children in low- and middle-income countries (LMICs) and are associated with a large burden of disability and mortality. There are limited data on the comorbidity of neurological conditions with behavioural and emotional problems. This study showed that neurological conditions are significantly associated with mental health problems in school-aged children and have long-term negative impact on these children. Cost-effective interventions are needed in LMICs to reduce the burden of these disorders.
