# Supplementary material for: Behavioural and emotional comorbidities in school-aged children with neurological conditions in Kilifi, Kenya, and their long-term consequences
Source: Glob Health Action. 2022 Feb 9;15(1):2034132. doi: 10.1080/16549716.2022.2034132 (PMC8843098; doi:10.1080/16549716.2022.2034132)
Supplement: Supplemental Material [file ZGHA_A_2034132_SM4920.docx]

**Supplementary material**

**Figure S1. Flow diagram on assessment of children for neurological conditions, mental health problems and follow-up**

10,218 children aged 6-9 years old assessed using TQQ for neurological conditions in 2001

Stage I: 9,263 (90.7%) TQQ negative

Stage I: 955 (9.3%) TQQ positive

Stage II: 716 (75.0%) clinically examined for neurological conditions

Stage II: 900 (9.7%) clinically examined for neurological conditions

393 (54.9%) assessed for long-term impact of neurological conditions, behaviour, and emotional problems in 2008 through the KHDSS

515 (57.2%) assessed for long-term impact of neurological conditions, behaviour, and emotional problems in 2008 through the KHDSS

323 (45.1%) lost to follow-up

385 (42.8%) lost to follow-up

Stage II: CBQFP administered to caregivers of 716 (75.0%) children to assess behaviour and emotional problems

Stage II: CBQFP administered to caregivers of 900 (9.7%) children to assess behaviour and emotional problems

**Table S1. Definition of different neurological conditions**

| **Domains of neurological impairment and epilepsy^*^** | **Moderate** | **Severe** |
| --- | --- | --- |
| Cognitive^a^ | Z-score of below −2 on two or more of the cognitive assessments OR mean Z-score for all tasks below −2 for a child who had performed construction task and a non-verbal task but not the key verbal tasks (picture vocabulary and information questions tasks) OR the mean Z-score for verbal tasks was below −2 | Z-score below −3 on two or more of the cognitive assessments OR mean Z-score below −3 for a child who had performed construction task and a non-verbal task but not the key verbal tasks (picture vocabulary and information questions tasks) OR the mean Z-score for verbal tasks was below −3 |
| Epilepsy^b^ | Frequency of seizures of once per month or greater | Frequency of seizures of once per week or greater |
| Hearing^b^ | 41- to 70-dB loss in the best ear and difficulty in hearing even with a hearing aid | 71 dB or more loss in the best ear, no useful hearing |
| Vision^b^ | Presenting distance visual acuity worse than 6/18m but equal to or better than 6/60m | Presenting distance visual acuity worse than 6/60m |
| Motor^b^ | Difficulty in holding objects, dressing, and sitting upright, able to move around with help | Inability to walk and absence of functional use of hands |

^a^Definitions adopted from Mung'ala-Odera *et al* [[1](#_ENREF_1)]

^b^Definitions adopted from the WHO classification manuals [[2](#_ENREF_2), [3](#_ENREF_3)]

**Table S2. Prevalence of mental health problems in children by age and sex**

| **Characteristic** | **Prevalence by type of mental health problem**  **(95% CI)** | | |
| --- | --- | --- | --- |
|  | **Total behavioural and emotional problem**  **(n=247)** | **Internalizing problem**  **(n=273)** | **Externalizing problem**  **(n=337)** |
| **Age of participants** | | | |
| 6 years | 21.7%  (18.0-25.4) | 19.4%  (15.9-22.9) | 24.2%  (20.3-28.0) |
| 7 years | 14.8%  (11.4-18.2) | 19.3%  (15.5-23.0) | 22.5%  (18.5-26.5) |
| 8 years | 12.2%  (8.8-15.6) | 15.8%  (12.0-19.6) | 19.7%  (15.5-23.8) |
| 9 years | 10.1%  (6.9-13.3) | 11.6%  (8.2-14.9) | 15.4%  (11.5-19.2) |
| **Sex** | | | |
| Male | 18.6%  (15.9-21.2) | 17.8%  (15.2-20.4) | 24.2%  (21.3-27.2) |
| Female | 11.9%  (9.6-14.1) | 15.9%  (13.4-18.5) | 17.3%  (14.7-19.9) |

**Table S3. Multivariable analysis of factors associated with total mental health problems, internalizing and externalizing problem scores**

| **Risk factor** | **Total behavioural and emotional problems scores** | | **Internalizing problems and scores** | | **Externalizing problems and scores** | |
| --- | --- | --- | --- | --- | --- | --- |
|  | **Adjusted multivariable**  **OR (95% CI)** | **Adjusted multivariable β coefficient**  **(95% CI)** | **Adjusted multivariable**  **OR (95% CI)** | **Adjusted multivariable β coefficient**  **(95% CI)** | **Adjusted multivariable**  **OR (95% CI)** | **Adjusted multivariable β coefficient**  **(95% CI)** |
| Neonatal jaundice | 2.28^**^ (1.38-3.75) | 2.19^**^ (1.47, 2.91) | 1.31 (0.78-2.20) | 0.35^**^ (0.08, 0.62) | 3.66^**^ (1.78-7.55) | 0.55 (-0.08, 1.18) |
| Number of children born to the family | - | 0.04 (-0.04, 0.11) | 1.06 (0.99-1.13) | 0.02 (-0.01, 0.05) | - | 0.05 (-0.01, 0.10) |
| Father’s lack of economic activity | - | - | - | 0.03 (-0.13, 0.18) | - | - |
| Mother’s lack of economic activity | - | - | 1.07 (0.78-1.47) | 0.01 (-0.14, 0.16) | 1.20 (0.75-1.93) | - |
| Single maternal marital status | 0.84 (0.55-1.27) | -0.24 (-0.71, 0.22) | - | - | 0.93 (0.50-1.70) | - |
| No maternal education | 0.63^**^ (0.44-0.89) | - | 0.63^*^ (0.45-0.87) | -0.17 (-0.32, 0.02) | 1.09 (0.68-1.76) | - |
| Home delivery | 0.95 (0.60-1.49) | - | - | - | 0.52^*^ (0.29-0.91) | - |
| Birth difficulty | 1.58 (0.93-2.69) | - | 1.58 (0.97-2.57) | 0.35^**^ (0.09, 0.60) | - | - |
| Neonatal insults | - | - | - | - | - | 0.18 (-0.33, 0.68) |
| Child not immunized | - | - | - | - | 1.55 (0.75-3.24) | - |
| Neurological deficit | 2.72^**^ (1.27-5.81) | 0.92 (-0.09, 1.94) | 2.31^*^ (1.15-4.63) | 0.41^*^ (0.04, 0.78) | 3.30^**^ (1.27-8.58) | 0.54^**^ (0.10, 0.97) |
| Developmental problems | 1.49 (0.97-2.32) | 0.42 (-0.12, 0.96) | 1.15 (0.75-1.74) | 0.16 (-0.05, 0.36) | 1.71 (0.95-3.06) | - |
| Cognitive impairment | 1.04 (0.59-1.81) | 0.36 (-0.34, 1.06) | 0.71 (0.41-1.24) | 0.10 (-0.16, 0.37) | 1.33 (0.64-2.73) | - |
| Hearing impairment | 1.04 (0.49-2.17) | - | 1.25 (0.64-2.45) | - | 0.47 (0.16-1.44) | - |
| Visual impairment | - | - | - | - | - | - |
| Motor impairment | 0.66 (0.27-1.64) | 0.11 (-1.09, 1.32) | 1.27 (0.56-2.86) | -0.08 (-0.52, 0.35) | 0.30 (0.08-1.11) | - |
| Epilepsy | 3.32^**^ (2.05-5.38) | 1.21^**^ (0.48, 1.93) | 2.03^**^ (1.26-3.26) | 0.43^**^ (0.16, 0.70) | 2.87^**^ (1.45-5.68) | - |

^*^p-value<0.05; ^**^p-value<0.01

**References**

1. Mung'ala-Odera V, Meehan R, Njuguna P, Mturi N, Alcock KJ, Newton CR. Prevalence and risk factors of neurological disability and impairment in children living in rural Kenya. International journal of epidemiology. 2006;35(3):683-8. Epub 2006/02/24. doi: 10.1093/ije/dyl023. PubMed PMID: 16492712.

2. Organization WH. International classification of impairments, disabilities, and handicaps: a manual of classification relating to the consequences of disease, published in accordance with resolution WHA29. 35 of the Twenty-ninth World Health Assembly, May 1976: World Health Organization; 1980.

3. Organization WH. ICD-11 for Mortality and Morbidity Statistics: World Health Organization; 2021 [updated 05/2021; cited 2021 05/07/2021]. 05/2021:[Available from: <https://icd.who.int/browse11/l-m/en>.
